# Control of parallelized bioreactors I: dynamic scheduling software for efficient bioprocess management in high-throughput systems

**DOI:** 10.1007/s00449-022-02798-6

**Published:** 2022-10-18

**Authors:** Lukas Bromig, Nikolas von den Eichen, Dirk Weuster-Botz

**Affiliations:** Chair of Biochemical Engineering, Technical University of Munich, Boltzmannstraße 15, 85748 Garching, Germany

**Keywords:** Parallel bioreactors, Dynamic scheduling, High-throughput systems, Automation, Device integration

## Abstract

The shift towards high-throughput technologies and automation in research and development in industrial biotechnology is highlighting the need for increased automation competence and specialized software solutions. Within bioprocess development, the trends towards miniaturization and parallelization of bioreactor systems rely on full automation and digital process control. Thus, mL-scale, parallel bioreactor systems require integration into liquid handling stations to perform a range of tasks stretching from substrate addition to automated sampling and sample analysis. To orchestrate these tasks, the authors propose a scheduling software to fully leverage the advantages of a state-of-the-art liquid handling station (LHS) and to enable improved process control and resource allocation. Fixed sequential order execution, the norm in LHS software, results in imperfect timing of essential operations like feeding or Ph control and execution intervals thereof, that are unknown a priori. However, the duration and control of, e.g., the feeding task and their frequency are of great importance for bioprocess control and the design of experiments. Hence, a software solution is presented that allows the orchestration of the respective operations through dynamic scheduling by external LHS control. With the proposed scheduling software, it is possible to define a dynamic process control strategy based on data-driven real-time prioritization and transparent, user-defined constraints. Drivers for a commercial 48 parallel bioreactor system and the related sensor equipment were developed using the SiLA 2 standard greatly simplifying the integration effort. Furthermore, this paper describes the experimental hardware and software setup required for the application use case presented in the second part.

## Introduction

New challenges arise with the advances in high-throughput technologies and the parallelization and miniaturization of bioreactor systems. Miniaturization is the means to achieve high-throughput bioreactor systems by parallelizing mL-scale bioreactors in liquid handling stations [1–6]. The miniaturization of bioprocesses supports the early stages of development by providing fast and cost-effective solutions for scale-up and process optimization studies [6]. The authors have shown in previous work that these systems provide a powerful toolset for bioprocess optimization by conducting fast protein expression studies [4]. However, deviations in process behavior due to the change of scale must be minimized and accounted for to ensure scalability of the experimental results to L- or pilot-scale reactors [7–10]. A good overview of current parallel bioreactor technology is given by Achinas et al. and Junne et al. [11–13].

Automated bioreactor control in liquid handling stations poses several challenges, as there are multiple parallel tasks that need to be performed to provide the same process conditions compared to the L-scale, such as pH-control and substrate addition. Alternate approaches directly translate the techniques from the L-scale to the miniaturized mL-scale by incorporating microfluidic systems into small, often disposable parallel reactor systems [14]. This leads to a labor-intensive and expensive solution that does not leverage the flexibility and power of liquid handling stations [11, 15].

By integrating the bioreactor system on the deck of a liquid handling station (LHS), these tasks can be handled by the pipetting station, which also greatly increases the types of operations that can be performed during the fermentation such as sample preparation and analysis [16]. However, this strategy can lead to differences regarding scalability in comparison to conventional L-scale benchtop bioreactors, because the execution of multiple tasks and their execution frequency is limited by the availability of the LHS pipetting channels. This results in a bottleneck problem that requires a scheduling solution.

Using an LHS for substrate addition limits the possible feeding strategies to intermittent feeding [4, 8, 17, 18]. As a result of the aforementioned bottleneck the minimum time interval at which feeding operations can be performed is restricted. This lower bound is defined by the speed of the liquid handling station, the number of parallel operations it can perform, the deck layout of the setup (i.e., distance to travel) and the number of tasks to be executed simultaneously. Other technical constraints of the LHS include the pipetting volume and modes, as well as the number of parallel channels. While many of those can be improved by upgrading the hardware or by a change of deck layout, a major contribution towards the mitigation of this problem depends on the execution order of the required tasks and the optimization thereof especially in bottleneck situations in which sampling and feeding tasks overlap.

Introducing dynamic scheduling algorithms into the proprietary software of a LHS is a cumbersome task, as the software has not been designed to handle such specific use cases. Regular implementations usually consists of fixed events or repeated, sequential execution of tasks. Thus, a dedicated software is required that is either implemented directly in the LHS software or implemented as an external software that controls the LHS. The software fedbatchXP (DASGIP, an Eppendorf SE company, Jülich) was aimed to solve bioprocess control with dynamic task scheduling and has been employed in past publications [1, 2, 7, 15, 19–21]. However, the software has been discontinued. Among others, the main drawbacks of this software were its proprietary, compiled nature, which made it impossible to freely customize and extend the software, as well as the intransparent nature of the prioritization algorithm. Furthermore, the software was designed for a specific device setup, a bioREACTOR48 integrated in a Tecan Freedom Evo, and was not open for integration of more or other devices, or further future development.

External control of liquid handling stations is gaining importance as laboratory infrastructure is becoming more digitized and processes more sophisticated. The authors propose a data-driven, dynamic scheduling solution with external LHS control. The software re-evaluates priorities of user-defined tasks in real time and orchestrates their execution for bioprocess control of a miniaturized and parallelized bioreactor system.

Use cases for the proposed dynamic scheduling software are manifold and software solutions that simplify the complexity of automated miniaturized bioprocess systems are required not just in industry, but academia as well [22]. Due to the complex nature of biological experiments, it is difficult to predict the changes in pH or the effect of different feeding strategies in advance. Conventional LHS software is designed to build fixed, sequential process workflows. However, the knowledge required to design a suitable execution sequence is not available a priori and rather the outcome of successful process development. Furthermore, sequential execution can lead to severe problems when tasks cannot be performed on time. With a flexible execution order, such tasks are performed on demand and not just on schedule.

An example area is general process optimization and the research on population heterogeneity in the scale-up of fermentation processes [23–25]. Deviations from ideal reactor behavior, such as changing levels of dissolved oxygen due to varying substrate availability resulting from intermittent feeding, have been linked to population heterogeneity [26, 27]. Moving between scales, non-ideality may vary in kind and magnitude, resulting in differences in product, metabolite, or inhibitor concentration or process variables like optimal harvesting time to achieve high space–time yield [28]. The proposed scheduling software enables researchers to further investigate and quantify these deviations by setting defined feeding intervals. If sequential execution was applied, the interval would be undefined a priori. Furthermore, the investigation of multiple intervals during a single run is enabled, while maintaining good process control of pH and the acquisition of frequent samples. Hence, the proposed software can speed up the screening process on a small scale.

Dynamic scheduling can improve process control as it remains flexible in regard to the execution order, but at the same time retains a stable and defined average execution interval. By re-evaluating sensor data and recalculating priorities of the individual tasks, an improved resource allocation can be achieved. The authors propose a software solution that consist of multiple separate services: a dynamic scheduler (LHS Scheduler), a broker that enables external control over the Liquid handling station (LHS Server), a digital twin (LHS Simulator) that can be used to emulate the LHS for *in silico* tests, as well as SiLA 2 device drivers for the bioreactor system and the related sensor equipment.

## Hardware and integration

The presented solution relies on the integration of multiple devices from different vendors. To overcome the barriers of varying proprietary device interfaces the standard *Standardization in Laboratory Automation* (SiLA 2) is used [29–31]. The SiLA standard is based on a server–client architecture and uses the remote procedure call protocol gRPC for communication, which is based on the standards HTTP/2 and protocol buffers. The developed software interfaces, i.e., SiLA Servers, for the parallel bioreactor system and the pH/DO sensors are built according to this standard.

### Parallel stirred-tank bioreactor system

The 48 × parallel stirred-tank bioreactor system (bioREACTOR 48, 2mag AG, Munich, Germany) is used for microbial fermentations on the mL-scale. The parallel bioreactor system was integrated into the software environment using the SiLA 2 standard. A SiLA Server was developed to translate the proprietary serial communication protocol (RS-232) of the hardware to provide the digital interface in the local network environment. The scheduler software controls the parallel bioreactor system with this SiLA 2 interface by a SiLA 2 client. Available functions include the starting and stopping of the reactor agitation, changing of the applied power, as well as the rotational speed settings. Furthermore, the interface can be used to obtain status data of the in-built Hall sensors, enabling the scheduler software to monitor and react to individual stirrer malfunctions. A simulation mode providing realistic data on, e.g., stirrer malfunctions was integrated to allow for *in silico* testing of the scheduler software. The SiLA 2 Server of the bioREACTOR48 was created using the SiLA 2 Python reference implementation [32] and is available as open-source project [33].

### pH/DO sensor bars

Dissolved oxygen (DO) and pH of the 48 bioreactors is measured in parallel by 6 fluorometric reader bars with 8 sensors each (MCR, PreSens GmbH, Regensburg, Germany), which are placed in a compartment underneath the mL-scale bioreactor vessels [5]. The developed PreSens SiLA Server standardizes the proprietary serial communication protocol and allows automated data acquisition of process parameters by the scheduler software via the local network. The PreSens SiLA 2 Server includes a simulation mode, which provides mock data for testing purposes. The SiLA 2 Server of the PreSens sensor bars was implemented using the SiLA 2 Python reference implementation [32] and is available as open-source project [33].

### Liquid handling station

A liquid handling station (Microlab® STARlet, Hamilton Bonaduz AG, Bonaduz, Switzerland) with eight 1000 μL pipetting channels is used to perform tasks on the 48 × parallel bioreactor system for the automated cultivation of microorganisms. The LHS is equipped with a microtiter plate (MTP) reader (Synergy HTX, BioTek, Winooski, USA) and a MTP washer (405 LS, BioTek, Winooski, USA), which are directly integrated into the hardware setup as periphery devices accessible by the LHS plate handler tool (iSWAP®, Hamitlon Bonaduz AG, Bonaduz, Switzerland).

A software package supplied by the vendor includes a method editor and a runtime environment (Microlab® STAR Software VENUS version 4.5, Hamilton Bonaduz AG, Bonaduz, Switzerland). Within the method editor, a user-friendly environment with method libraries and drivers for periphery devices is provided, which were used to integrate the MTP washer and reader. However, more complex methods or foreign device integrations require the use of the underlying vendor-specific Hamilton Standard Language (HSL) or custom support. Due to the number of external devices to be integrated and the complexity of a dynamic prioritization algorithm, a solution written in HSL was not an option and a method had to be found to take control of the LHS externally through the scheduling software. The device was integrated using the COM-Interop interface of the vendor software and a newly developed gRPC server. The gRPC server acts as a broker and enables external control of the LHS via the local network. A more detailed account of the external control is explained in the LHS Server section.

### Example application setup

**Fig. 1:**
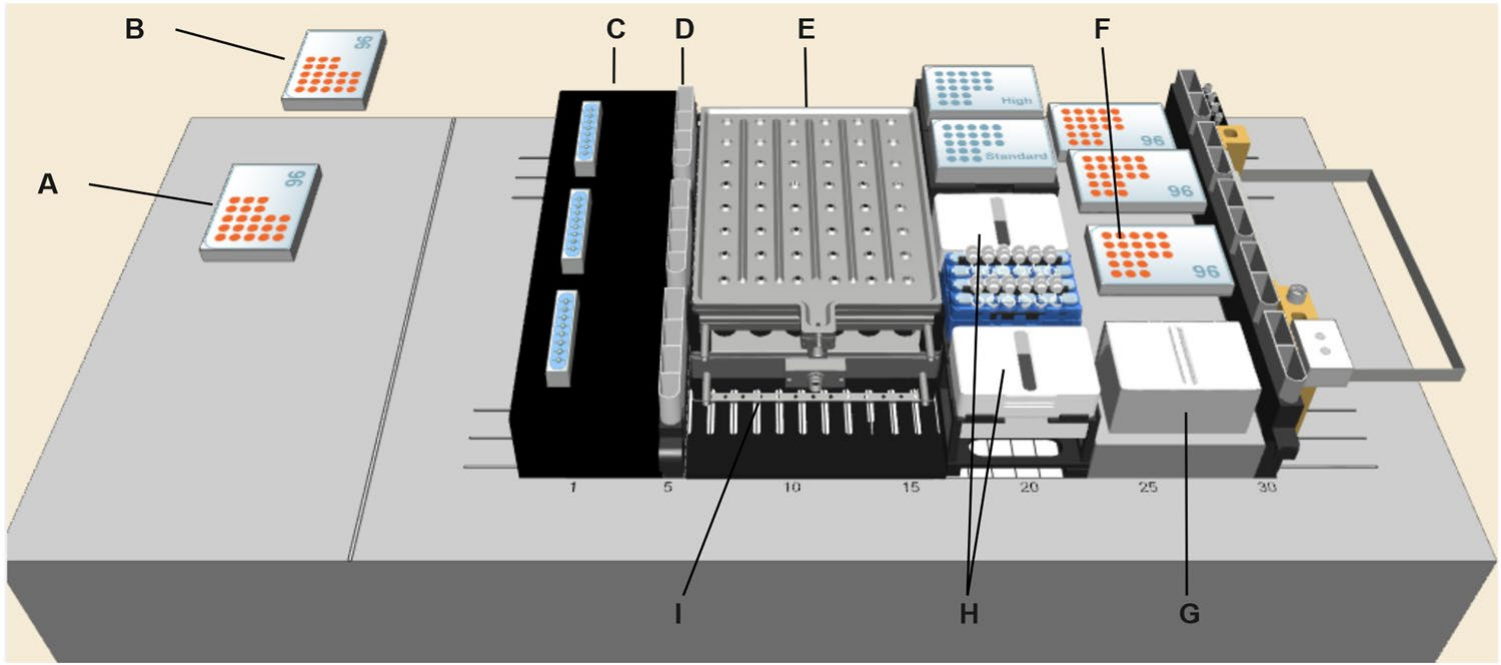
A 3D visualization of the LHS deck layout showing the accessible entities and their positions as shown by the control software VENUS Run Control. From left to right, the setup consists of a MTP washer (**A**), a MTP reader (**B**), a pipetting needle wash station (**C**), several containers with aqueous EtOH (70% v/v) for pipetting needle disinfection (**D**), a bioREACTOR48 (**E**), a MTP for sample de-aeration, dilution and preparation (**F**), a container for substrate storage (**G**), containers for pH-controlling (NaOH) and phosphate buffered saline (**H**), and the socket of the pH/DO sensor bars (**I**, bars not shown). The plate reader and plate washer are accessed by the integrated plate gripper (iSWAP™)

The target applications of the LHS Scheduler are microbial cultivations in the miniaturized bioreactor system BioREACTOR48 which is integrated into a liquid handling station. Figure 1 shows the setup for which the software has been developed and tested.

## Software architecture and implementation

### General software architecture

**Fig. 2:**
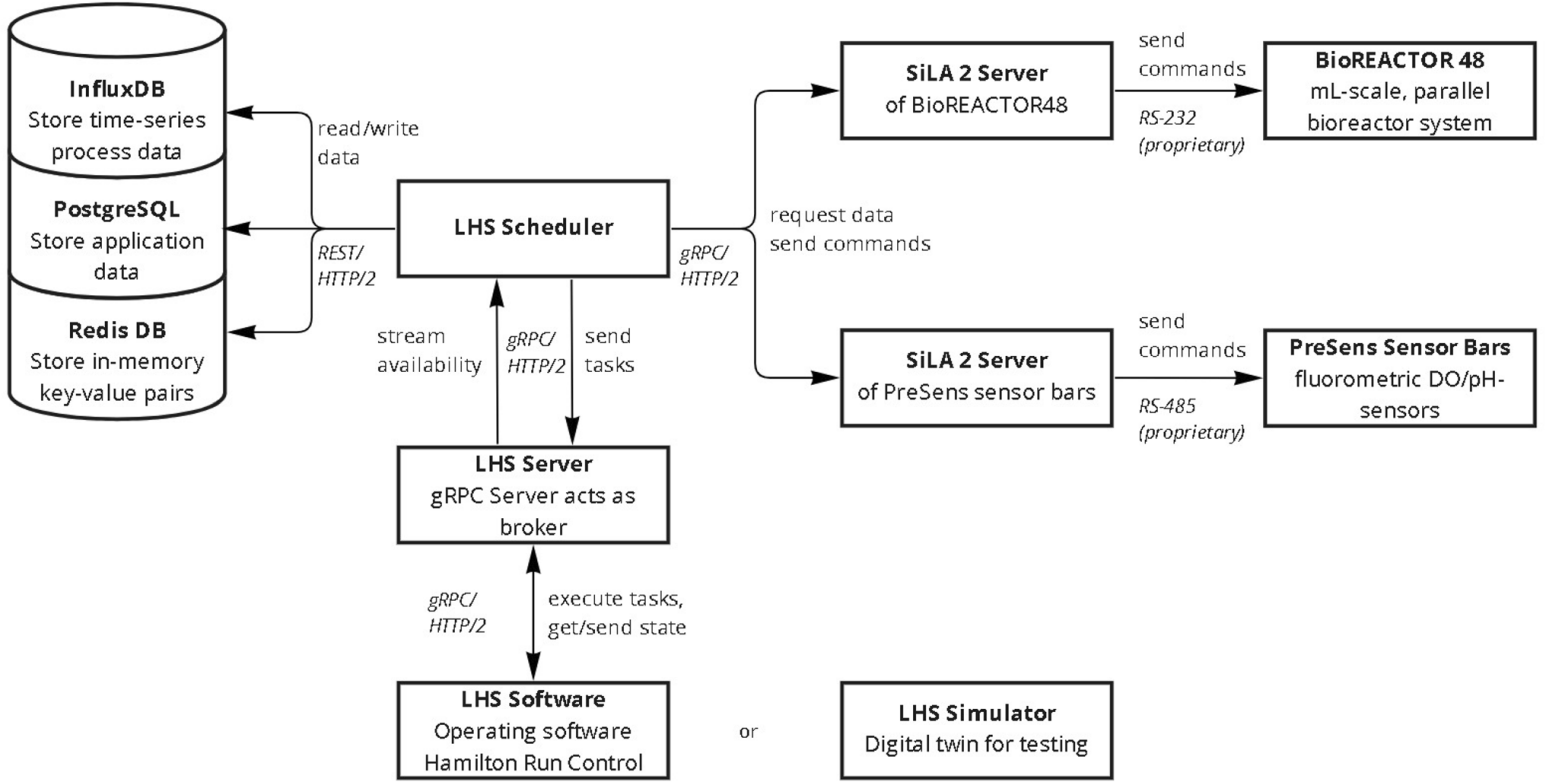
The proposed software solution consists of several elements: The central scheduler application, the SiLA 2 device servers for the parallel bioreactor system and the pH/DO sensor bars, the gRPC server for LHS communication. Furthermore, it relies on three databases for the storage of experiment process data (InfluxDB), the persisting of application data (PostgreSQL) and for in-memory storage and inter-thread and process communication (RedisDB)

The software solution consists of multiple services, which are connected to the central scheduler application as shown in Fig. 2. The reactor system and the pH/DO sensors are connected to the LHS Scheduler by a standardized SiLA 2 server–client connection, whereas the integration of the LHS is realized through a gRPC broker server, which evaluates, processes, and forwards traffic between the scheduler application and the LHS. Furthermore, the scheduler application is connected to two databases to persist process and application data.

#### InfluxDB

InfluxDB (InfluxDB v.1.7.11, InfluxData, San Francisco, USA) is an open-source NoSQL time-series database [34, 35]. The scheduler application uses InfluxDB to store the acquired process data that is received from the external devices in real time. This includes data of the bioreactor system, the sensor bars, the LHS, as well as the evaluated and processed data such as task priorities, task execution times, and setpoints. The stored data are enriched with timestamps and meta data such as experiment name, reactor position, and operator to enable good data management and data filtering for, e.g., real-data visualization or data export for subsequent analyses. The LHS Scheduler uses the python package *influxdb* to communicate with the database server. If no dedicated InfluxDB server is provided to the application, a fall-back database will be created using the official InfluxDB image hosted on DockerHub (*influxdb* v.1.7.11). Process data are visualized in real time using the complimentary web-service application Chronograf (Chronograf v.1.8.5, InfluxData, San Francisco).

#### PostgreSQL

PostgreSQL (PostgreSQL v.6.0.9, PostgreSQL Global Development Group) is a widely adopted SQL database. The python package *psycopg2* (v.2.8.6) is used to communicate with the database from within the LHS Scheduler application. If no dedicated PostgreSQL server is available, a fall-back database is created. This database is run in a docker container using the official PostgreSQL docker image available on dockerhub (*postgres* v.13). The complimentary database management system pgAdmin4 (pgAdmin4 v.5, pgAdmin Development Team) is used for data access and visualization of the application data.

#### LHS server

**Fig. 3:**
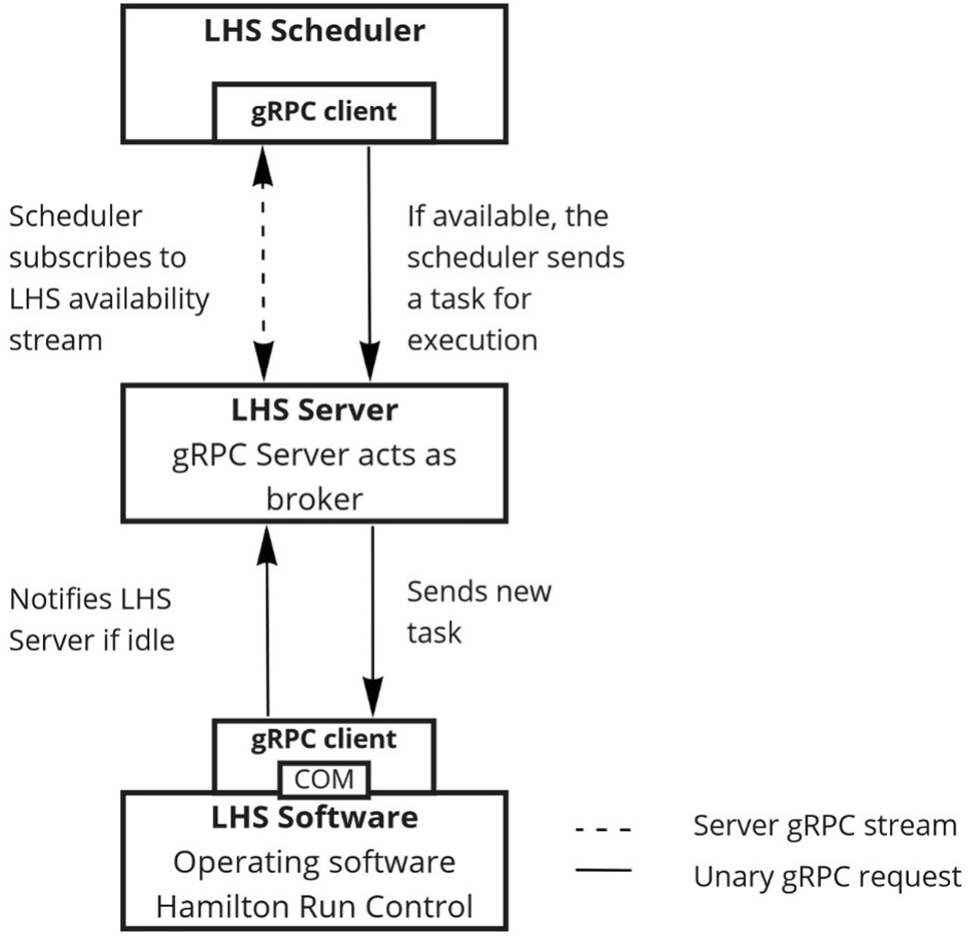
External control of the LHS is realized by introducing a C# gRPC client into the VENUS environment by registering it as COM-Interop and including it into the VENUS method library. The dedicated LHS gRPC Server acts as broker between the scheduling software and the VENUS runtime. The scheduler incorporates a python gRPC client to communicate with the gRPC server, hence controlling the LHS by sending execution request and receiving the responses transferred by the LHS Server. At the same time, the LHS Server is aware of the LHS’s state and shares this information with the scheduler software through a gRPC stream. This leads to an event-based execution with minimal delay and downtime

Pipetting stations are common island solutions and integration into other third-party software can be difficult as software interfaces are oftentimes missing, hidden, or unexposed. In most cases, external control is not required or not intended. For the proposed scheduling software however, enabling external control is a critical requirement. HSL, deriving from C, supports the execution of code elements that were registered on the operating system as Component Object Model (COM). COM interop is a technology developed by Microsoft to enable interoperability between COM-libraries and the Windows.NET Framework in the Common Language Runtime (CLR) [36, 37]. This allows the registration of C# code to be registered as COM-Interop and subsequently to be imported into the VENUS method editor library. With this procedure, an interface was created based on a C# gRPC client that was introduced into the VENUS environment as shown in Fig. 3.

The information transmitted between the LHS Scheduler and the LHS runtime environment via the LHS Server is kept as simple as possible including only the essential information required for execution. This encompasses the name of the method, an ordered array containing the volumes to be transferred, as well as the respective target and, if required, source sequence. Target and source sequences are transferred as lists (Python) and arrays (C#) and are mapped to the labware definition within Venus. This ensures that only existing positions are addressed.

#### LHS simulator

To improve the development process and increase the software quality, a digital twin of the LHS has been developed to mimic the behavior for *in silico* software testing: The LHS Simulator. The LHS simulator receives commands from the LHS Server, interprets and checks their validity regarding constraints and execution order, and simulates the execution time based on historical data. The LHS simulator loads the user script at the start of a simulation run and evaluates the specifications supplied by the user. During runtime, the scheduler output is compared against the user input. Discrepancies between the specifications in the user script and the output regarding parameters like violated constraints, missed executions, or miscalculated feeding volumes are stored are detected and stored in a log file. The LHS Simulator is based on a gRPC Client and uses the same interface description as the C# client of the LHS. The source code is written in python.

## Dynamic scheduling software

### The scheduler core

The LHS Scheduler is based on a gRPC server. Once started, the application is configured by an experiment-specific user script. Within this user script, the information about the experiment, the used time-series database, the involved devices, and the tasks and their respective configuration is provided. This information is persisted in the PostgreSQL database as application data via invocation of the corresponding function of the application programming interface (API). The API is a useful tool for testing and development as it allows the interaction with the scheduler application during runtime. However, this is not required nor intended for the regular use case presented in Control of Parallelized Bioreactors II.

A scheduler instance consists of three phases: initialization, run, and termination. During initialization, the information on the experiment, the specified tasks, the required devices and databases is loaded. At this stage a device initialization sequence is run that sets up a device connection and configures them correctly for use by the application. At the end of the initialization procedure, threads for data acquisition and the initially scheduled tasks are started.

During a fermentation process, the scheduler application continuously communicates with the task threads and keeps track of their reported priorities and status. Furthermore, the data acquisition threads are monitored and restarted if needed. Within the run-loop, tasks can be added or removed from the scheduler, which will spawn or kill the respective task threads. Furthermore, the run-loop subscribes to a stream provided by the LHS Server, to keep track of the status of the LHS. The availability of the LHS is communicated via this stream in real time. If available, the scheduler will dispatch the task with the highest priority to the LHS Server for execution. The scheduler instance and the task threads all rely on the internal clock of the scheduler instance main loop. For simulation purposes, the internal time can be manipulated for fast run simulations. In this mode, the LHS Simulator is used and the device drivers of the bioreactor system and the sensor bars are set to simulation mode.

The termination sequence is a safety mechanism that is executed in the following two scenarios: (1) The scheduler shuts down in a controlled way either by invoking the respective API command or by the execution of a scheduled abort task or (2) if the scheduler main loop exits unexpectedly. The termination sequence stops all running task threads, closes the stream channel with the LHS Server, stops the device data acquisition, and executes a device termination sequence. This is particularly important, if devices are used that may take or cause damage in case of an unexpected loss of control. The device termination sequence will break out of the run-loop and resume in the LHS Scheduler main loop.

### Data acquisition

The dynamic scheduling capability is dependent on the availability of real-time data. Before entering the scheduler run-loop, the LHS Scheduler starts a data acquisition thread for each data source. The data sources are defined in the user script and, thus, the API. A data source is defined by their SiLA 2 connection details, the measurement intervals and, similar to the scheduler run-loop, respective data acquisition scripts for initialization, run, and termination. The data is stored in the InfluxDB and accessible to the other threads for processing and priority calculation.

**Fig. 4:**
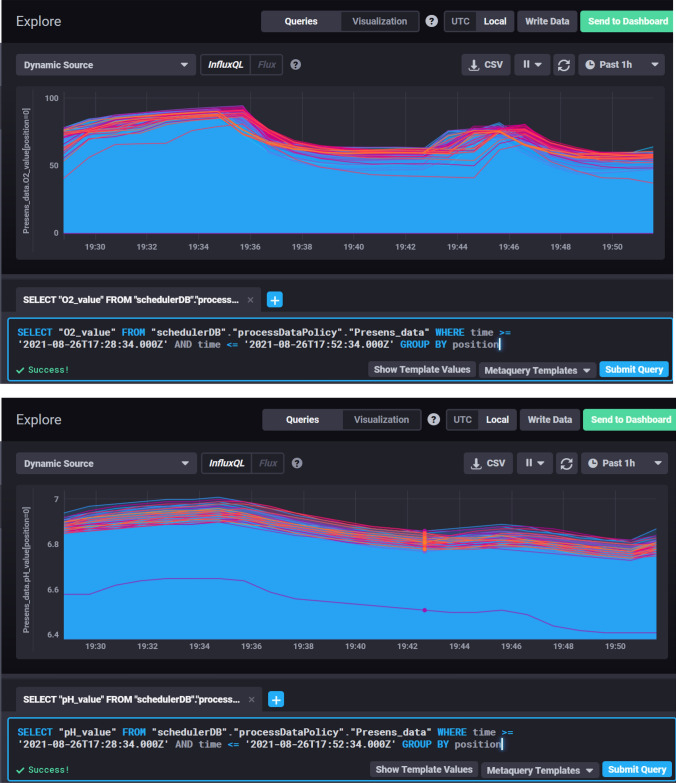
The browser-based graphical user interface Chronograf connects to the InfluxDB and allows for real-time data visualization. In this figure, the process parameters dissolved oxygen (top) and pH (bottom) are shown for the same excerpt of an example process in parallel stirred-tank bioreactors

The process data can be accessed either directly by using an InfluxDB database client or the web-based visualization tool Chronograf as shown in Fig. 4. The LHS Scheduler application and its task and data acquisition threads write their data into the InfluxDB database. Each data point consists of a measurement name, tags like experiment name and reactor positions, a timestamp, and fields containing the values of the parameters that make up that measurement. A data point is unique. Specific data points or subsets thereof can be visualized or exported using the SQL-like query language InfluxQL as shown in Fig. 4.

**Fig. 5:**
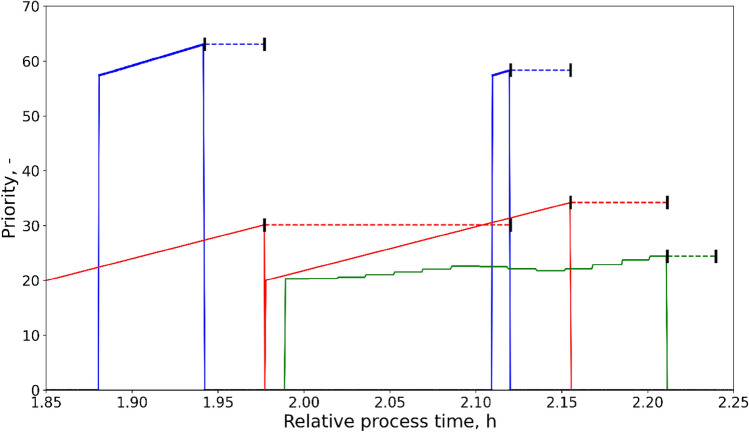
The priority changes of varying tasks are shown: the feeding task (−, blue line), stages 4 and 5 of the sampling task (−, red line), and the pH-control task (−, green line). The execution times of these tasks are marked by dashed lines in the respective color with bars representing the start and end time. The relative process time is identical to the example excerpts of the process data shown in Fig. 5. The substrate solution contained 300 g L^−1^ glucose. Cultivation conditions and process parameters are described in detail in the second part (Control of Parallelized Bioreactors II)

Apart from defining the database, the retention policy and the measurement of interest, the query can be filtered by tags, timestamps and a combination thereof. The queries in Fig. 4 fetch and visualize the dissolved oxygen (top) and pH (bottom) data of 48 reactor positions over the duration of 24 min of an *E. coli* fed-batch process described in Control of Parallelized Bioreactors II. The operations executed by the LHS at that time are displayed in Fig. 5.

### Task threads

To run fermentation experiments in mL-scale bioreactors that are integrated into LHS systems, several tasks must be performed by the LHS. These tasks include, but are not limited to, substrate addition, pH control, induction, and a sampling task that can consist of multiple sub-steps (for example: sampling, sample preparation, plate reader measurement, cleaning of MTP). The LHS Scheduler executes the task with the highest priority. Priorities are calculated by the tasks threads themselves based on repeated evaluation of the available real-time data, such as the current pH in every reactor at the current time. A task for DO-control utilizing the available real-time DO data and the access to the bioreactor stirrer was not implemented as DO-control was not required by the application use case presented in the second part (Control of parallelized bioreactors II).

Task threads and their specific implementation may vary between use cases. To allow for the creation of new tasks or enable adaptation of existing tasks, the task thread class inherits from a base class that implements all functions that are shared between tasks.

Running tasks threads communicate with the LHS Scheduler run-loop via queue objects to report their current priority. If the scheduler selects the task for execution, the task thread is informed about the start- and end time of the event. Task thread data such as the thread runtime, the time of the last execution, the priority as well as set points and present values are written frequently to the influx database with the respective experiment and task-specific tags.

### Priority calculation

The execution of a task depends on external factors such as physical limitations and the design of the experiment like fixed execution times for an induction operation. These constraints must be evaluated before any priority calculation. Constraints are very task specific and require the in-depth knowledge of the process and the involved machines. Pipetting robots may, for example, not be able to pipette extremely small or large volumes due to physical limitations of the pipetting channels. Furthermore, a process with a substrate addition task must be performed on an interval that is itself constrained by upper and lower time boundaries so that the deviation from that interval remains minimal. Hence, there are time and volume constraints. While some of these constraints are soft and may be violated, such as an upper time constraint of a substrate addition task, some are hard constraints that prohibit execution, such as the minimum volume that can be pipetted.

Constraints are reactor specific, vary from task to task and must be specified by the user. A python class is provided that fully defines a constraint. A task thread will use the constraint evaluation function to assess each constraint and whether and to what extent, each constraint is violated and output a vector containing a boolean for each reactor. The task thread will resume with the priority calculations only if no hard constraints are violated.

All task thread objects use the same priority calculator class in which the priority calculation algorithms are defined. A task thread contains an instance of the priority manager and passes all relevant data to this object, such as the algorithm to be used and the number of active reactor positions. The number of tasks that require the shared resource, the dispensing unit, is low. However, the issue lies within the bottleneck situations in which multiple executions are necessary at the same time. This requires the optimization of the execution order based on priorities that are calculated based on real-time data. It was found that this can be achieved with simple static and linear functions as priority algorithms.

The priority calculator differentiates between no priority, a base priority and a critical priority. A task that either violates a hard constraint or does not require execution has a priority equal to zero. If the need for execution is evaluated, the priority calculation has access to the parameters ${p}_{\mathrm{base}}$ and ${p}_{\mathrm{crit}}$. The priority of a single task cannot exceed its critical priority:

$$p_{{{\text{max}}}} = p_{{{\text{crit}}}} .$$

The implemented priority calculation functions are:

#### Step

A static priority determination that depends on constraint evaluation alone. If a task is within its constraint limits, it is executable. As soon as it starts exceeding its soft boundaries, the priority will be raised.

$$p = \left\{ {\begin{array}{*{20}c} {0 , \quad \quad \quad {\text{if hard constraint violated}}} \\ {p_{{{\text{base}}}} ,\quad \quad \quad {\text{if no constraint violated}}} \\ {p_{{{\text{crit}} }} ,\quad \quad \quad {\text{if soft constraint violated}}{.}} \\ \end{array} } \right.$$

#### Step specific

The priorities are broken down to the relative contributions of the affected reactor positions that require action (${n}_{\mathrm{aff}})$ respective to the number of total active reactor positions (${n}_{\mathrm{all}})$.The number of total active reactors may vary as reactor positions with malfunctions are automatically excluded by the LHS Scheduler.

$$p\left( {n_{{{\text{aff}}}} , n_{{{\text{all}}}} } \right) = \left\{ \begin{gathered} 0, \quad\quad\quad{\text{if}}\;{\text{hard}}\;{\text{constraint}}\;{\text{violated}} \hfill \\ p_{{{\text{base}}}} \frac{{n_{{{\text{aff}}}} }}{{n_{{{\text{all}}}} }},\quad\quad\quad{\text{if}}\;{\text{no}}\;{\text{constraint}}\;{\text{violated}} \hfill \\ p_{{{\text{base}}}} + \left( {p_{{{\text{crit}} }} - p_{{{\text{base}}}} } \right)\frac{{n_{{{\text{aff}}}} }}{{n_{{{\text{all}}}} }},\quad\quad\quad{\text{if}}\;{\text{soft}}\;{\text{constraint}}\;{\text{violated}}{.} \hfill \\ \end{gathered} \right.$$

#### Dynamic volume

The priority is a linear function of the volume and the number of active positions and the contributions towards the priority are weighted per reactor positions. This function is aimed at tasks in which the execution depends on volume, such as the required volume of substrate in a feeding task or the volume of base to be added in a pH-control task. The vector $v$ contains the feed or base volumes of each reactor position that fulfills the constraints. The vector ${v}_{\mathrm{lim},\mathrm{ lower}}$ contains the lower volume limit that can be added for each position and ${\Delta v}_{\mathrm{lim}}$ contains the allowed volume space between the lower and upper volume limit of each position (${\Delta v}_{\mathrm{lim}}={v}_{\mathrm{lim},\mathrm{ upper} }-{v}_{\mathrm{lim},\mathrm{ lower}}$).

$$p\left( {n_{{{\text{all}}}} ,n_{{{\text{aff}}}} ,v } \right) = \left\{ \begin{gathered} 0,\;{\text{if}}\;{\text{hard}}\;{\text{constraint}}\;{\text{violated}} \hfill \\ p_{{{\text{base}}}} + \frac{{p_{{{\text{crit}}}} - p_{{{\text{base}}}} }}{{n_{{{\text{all}}}} }}\mathop \sum \limits_{i = 1}^{{n_{{{\text{aff}}}} }} \frac{{v_{i} - v_{i,\lim ,lower } }}{{\Delta v_{i, \lim } }},\quad\quad{\text{if}}\;{\text{no}}\;{\text{constraint}}\;{\text{violated}} \hfill \\ \end{gathered} \right..$$

#### Dynamic time

The priority is a linear function of the time and the number of active positions. The contributions of each reactor position are weighted. This function is aimed at tasks in which the execution is time dependent, such as sampling or induction. The vector $t$ contains the time since the last execution of each reactor position that fulfills the constraints. The remaining vector notation is transferable from the previous method.

$$p\left( {n_{{{\text{all}}}} ,n_{{{\text{aff}}}} ,t} \right) = \left\{ \begin{gathered} 0,\quad\quad {\text{if}}\;{\text{hard}}\;{\text{constraint}}\;{\text{violated}} \hfill \\ p_{{{\text{base}}}} + \frac{{p_{{{\text{crit}}}} - p_{{{\text{base}}}} }}{{n_{{{\text{all}}}} }}\sum\limits_{i = 1}^{{n_{{{\text{aff}}}} }} {\frac{{t_{i} - t_{{i,\lim ,{\text{lower}}}} }}{{\Delta t_{i,\lim } }}} ,\quad\quad{\text{if}}\;{\text{no}}\;{\text{constraint}}\;{\text{violated}}{.} \hfill \\ \end{gathered} \right.$$

Figure 5 displays the behavior in a bottleneck situation in which multiple tasks require access to the LHS. In this example, a simple linear dynamic priority calculation dependent on time (dynamic time: sampling) or volume (dynamic volume: feeding and pH control) was applied. As a result, the feeding task was executed with the minimal possible feeding interval. Furthermore, the last sampling step could be executed swiftly as pH regulation was not required urgently, with a, at times, even decreasing priority (also see pH visualization in Fig. 4). The decreasing priority of pH can be explained by a switch of metabolism in the *E. coli* cultures from overflow metabolism to glycogen metabolism [38]. The first substrate addition (Fig. 5) leads to overflow metabolism and acetate production, lowering the pH continuously (Fig. 4). Surpassing the threshold of the pH control, the lower volume constraint of base, the priority rises. The switch to glycogen metabolism and the corresponding consumption of acetate increases the pH and, thus, lowers the priority of the pH-control task.

## Conclusion and outlook

The proposed software enables the dynamic scheduling of LHS operations required for bioprocess control in parallel bioreactor systems, leading to an improved task execution order compared to conventional proprietary vendor software. The user can create tasks with pre-defined execution intervals allowing experiments in which these parameters are of utmost importance, like studies on scalability regarding protein expression or population heterogeneity, while relying on simple, transparent and editable priority calculation algorithms.

The acquisition of all process data by the LHS Scheduler software in a central database reduces the risk of information loss or error and enables real-time visualization of the data. Storing data of multiple sources in a central database makes the data readily available during the process and afterwards, simplifying data processing and analysis. The data generated in regular optimization processes is time-series data. However, if data sources such as chromatography data or data stemming from image analyses are to be acquired, time-series databases would not be well suited, as they are not optimized for such data types. In such cases, the setup of a data lake should be considered in future work.

The increasing degree of complexity of automated fermentation setups results in an increasing number of points of failure. As this is not desirable, testing of software and experimental plans is important. While software malfunctions can be prevented by unit, integration, and end-to-end tests, the actual simulation of experimental runs remains complicated when dealing with biological systems. As proposed, the execution of *in silico* experiments prior to the experimental run can be achieved by simulating experimental conditions with digital twins of the devices involved. Thus, future work should aim towards improving code quality by extending the existing testing infrastructure but also by improving the digital twin implementations of the bioreactor system, the sensor bars and the LHS to achieve more realistic *in silico* experiment simulations by, e.g., the integration of mechanistic models or the use of empirical data.

The LHS Server opens a backdoor to take external control of a Hamilton STARlet LHS and thus the integration of such systems into greater automation workflows. Other approaches aim towards full external control, thus transferring LHS responsibilities like tracking of lab ware positions to the user [39, 40]. This approach is error prone and complex because it requires intrinsic knowledge of the LHS as it circumvents the method editor supplied by the vendor. The proposed solution is a good compromise between the two, as it leverages the benefits of external control as well as the proprietary method editor.

The presented software consists of the LHS Scheduler, Server, and Simulator as well as the SiLA 2 device servers. They are written in Python, making them easily readable and adjustable to changing requirements. The general structure of the LHS Scheduler enables the expansion with new tasks or priority calculation algorithms. Future work should include the development of a graphical user interface to increase usability and enable human intervention in the running scheduling process, like adjusting task definitions during runtime.

The following software components are provided on request by the authors: LHS Scheduler, LHS Server, LHS Simulator. The SiLA 2 device drivers for the BioREACTOR48 and the Presens sensor bars are publicly available in the following GitLab repository: https://gitlab.com/biovt/sila2lib_implementations

## Data Availability

The SiLA 2 device drivers are publicly available in the following GitLab repository: https://gitlab.com/biovt/sila2lib_implementations
